# Identification of *cis*-regulatory modules for adeno-associated virus-based cell-type-specific targeting in the retina and brain

**DOI:** 10.1016/j.jbc.2022.101674

**Published:** 2022-02-09

**Authors:** Cheng-Hui Lin, Yue Sun, Candace S.Y. Chan, Man-Ru Wu, Lei Gu, Alexander E. Davis, Baokun Gu, Wenlin Zhang, Bogdan Tanasa, Lei R. Zhong, Mark M. Emerson, Lu Chen, Jun B. Ding, Sui Wang

**Affiliations:** 1 Department of Ophthalmology, Mary M. and Sash A. Spencer Center for Vision Research, Byers Eye Institute, Stanford University, Stanford, California, USA; 2 Department of Neurosurgery, Stanford University, Stanford, California, USA; 3 Epigenetics Laboratory, Max Planck Institute for Heart and Lung Research, Bad Nauheim, Germany; 4 College of Pharmaceutical Sciences, Zhejiang University, Hangzhou, China; 5 Department of Biology, The City College of New York, New York, New York, USA; 6 Department of Neurology and Neurological Sciences, Stanford University, Stanford, California, USA

**Keywords:** retina, brain, virus, glial cell, neuron, transcription factor, *cis*-regulatory module (CRM), AAVs, AAV, adeno-associated virus, AC, amacrine cells, AP, alkaline phosphatase, BP, bipolar, CNS, central nervous system, CRM, *cis*-regulatory module, GCL, ganglion cell layer, INL, inner nuclear layer, MG, Müller glial cells, PFA, paraformaldehyde, PR, photoreceptor, PV, parvalbumin, TFBS, transcription factor binding site

## Abstract

Adeno-associated viruses (AAVs) targeting specific cell types are powerful tools for studying distinct cell types in the central nervous system (CNS). *Cis*-regulatory modules (CRMs), *e.g.*, enhancers, are highly cell-type-specific and can be integrated into AAVs to render cell type specificity. Chromatin accessibility has been commonly used to nominate CRMs, which have then been incorporated into AAVs and tested for cell type specificity in the CNS. However, chromatin accessibility data alone cannot accurately annotate active CRMs, as many chromatin-accessible CRMs are not active and fail to drive gene expression *in vivo*. Using available large-scale datasets on chromatin accessibility, such as those published by the ENCODE project, here we explored strategies to increase efficiency in identifying active CRMs for AAV-based cell-type-specific labeling and manipulation. We found that prescreening of chromatin-accessible putative CRMs based on the density of cell-type-specific transcription factor binding sites (TFBSs) can significantly increase efficiency in identifying active CRMs. In addition, generation of synthetic CRMs by stitching chromatin-accessible regions flanking cell-type-specific genes can render cell type specificity in many cases. Using these straightforward strategies, we generated AAVs that can target the extensively studied interneuron and glial cell types in the retina and brain. Both strategies utilize available genomic datasets and can be employed to generate AAVs targeting specific cell types in CNS without conducting comprehensive screening and sequencing experiments, making a step forward in cell-type-specific research.

Molecular tools that allow for cell-type-specific labeling and manipulation in the central nervous system (CNS) are important for understanding how this complex tissue works and responds to disease. Adeno-associated viruses (AAVs), which can provide safe and long-lasting expression, offer a powerful way to target CNS cells in a rapid, cost-effective, and efficient manner (1, 2). Targeted AAV injections into different regions of the CNS have been widely used to label and study CNS cells and diseases (3, 4). The challenge is to develop recombinant AAV-based tools that allow for cell-type- or subtype-specific labeling and manipulation. One of the major strategies for developing such AAVs is to integrate *cis*-regulatory modules (CRMs) that can control cell-type- and stage-specific gene expression into AAV vectors. This strategy restricts the expression of genes carried by AAVs to specific cell types without changing the tropism of AAVs. Multiple methods have been developed to screen for cell-type-specific CRMs based on chromatin accessibility, genomic DNA methylation pattern, sequence conservation, and transcription factor binding sites (TFBSs) (5, 6, 7, 8, 9, 10). However, most of these methods rely on performing genome-wide sequencing, such as single-cell ATAC-seq (scATAC-seq), or large-scale screening. The efficiency of identifying cell-type-specific CRMs for AAVs has also not been high, especially for nonabundant cell types in the CNS. As many large-scale datasets on chromatin accessibility, *e.g.*, ENCODE project (11, 12), are available for CNS tissues, efficient strategies that utilize these datasets should significantly benefit the generation of cell-type-specific AAV tools. The neural retina serves as an excellent model system for developing such strategies. It is composed of more than a hundred distinct cell types/subtypes and can be easily accessed *via* surgical techniques (13, 14, 15, 16, 17). Using this model, we explored strategies that can efficiently identify CRMs for AAV-based cell-type-specific targeting and applied them to the brain.

We nominated putative CRMs based on chromatin accessibility as revealed by the ENCODE project and published ATAC-seq datasets for the retina and brain (12, 18). We performed a pilot study to search for common features of active CRMs and found that bioinformatically prescreening of chromatin accessible putative CRMs based on the density of cell-type-specific TFBSs can significantly increase the efficiency of identifying active CRMs. For cell types with limited information, generation of synthetic CRMs by stitching chromatin accessible regions flanking cell-type-specific genes can often straightforwardly render cell type specificity without conducting complicated screens. Using these strategies, we generated AAVs that target extensively studied CNS cell types, including Müller glial cells and amacrine interneurons in the retina, and astrocytes and parvalbumin (PV)-positive interneurons in adult brain.

## Results

### Cell-type-enriched TFBS density can be used to predict the activity of CRMs

**Figure 1:**
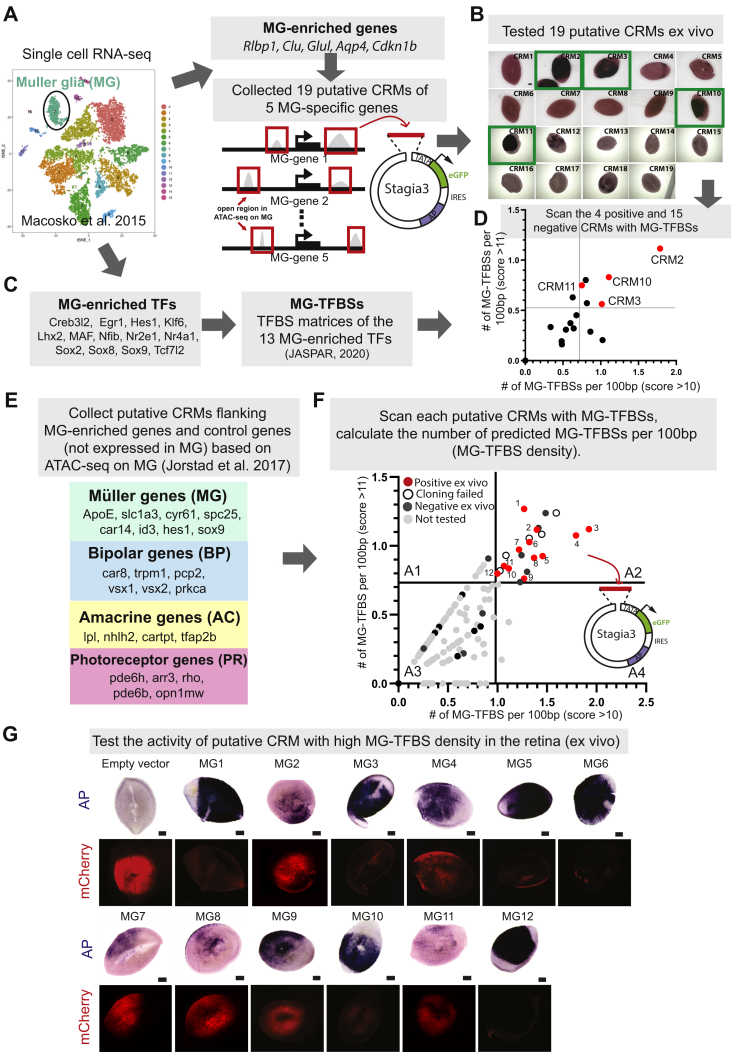
**The prescreening strategy for identifying cell-type-specific active CRMs using retinal Müller glial cell (MG) as a model system.***A*–*D*, the pilot study for extracting features that can distinguish active CRMs from inactive CRMs in retinal MG. *A*, based on single-cell RNA-seq data (17) and the literature, five genes that are known to be enriched in MG were selected. Nineteen chromatin accessible regions flanking these five MG-enriched were selected as putative CRMs based on ATAC-seq of purified mouse MG (18). We cloned each putative CRM into the Stagia3 vector (24). *B*, thirteen MG-enriched TFs were collected based on single-cell RNA-seq data (17). Their binding motifs (MG-TFBSs) were collected from the JASPAR database (45). *C*, each putative CRM reporter plasmid was electroporated into P1 mouse retinas and cultured for 5 days *ex vivo*. The activity of each putative CRM was indicated by AP (Alkaline Phosphatase) expression. *Green open box*, positive CRMs with strong activities. Scale bar: 50 μm. N ≥ 4 retinas. *D*, we scanned the DNA sequence of each putative CRM with MG-TFBSs using JASPAR database. The total number of MG-TFBSs per 100 bp (MG-TFBS density) for each putative CRM was plotted. X axis: the number of MG-TFBSs per 100 bp with JASPAR relative score >10 (the higher the score, the higher similarity between the sequence and JASPAR TFBS matrix). Y axis: the number of MG-TFBSs per 100 bp with JASPAR relative score >11. *Red*, positive CRMs; *Black*, negative CRMs. *E* and *F*, prescreening of putative CRMs using the hypothesis drawn from the pilot study, which showed that the MG-TFBS density is correlated with CRM activity. *E*, the MG-enriched genes and control genes (enriched in retinal bipolar, amacrine, and photoreceptor cells) were collected based on single cell RNA-seq data of adult mouse retina (17) and published literature. The putative CRMs flanking these genes were collected based on MG ATAC-seq (18). *F*, each putative CRM was scanned with MG-TFBSs (*B*) using JASPAR database. The number of MG-TFBSs per 100 bp was plotted as described in panel *D*. A1–A4: four quadrants of the plot. We cloned some putative CRMs in quadrant A1, A2 and A3 into reporter plasmid Stagia3 and tested their activities in the retina *ex vivo*. *Light grey dot*, untested putative CRMs; *Dark gray dot*, tested CRMs without any activities in the retina; *Red*, active CRMs in the retina. *Black circle*, CRMs failed to be cloned due to high GC contents or repetitive sequences. 1 to 12, MG1 to MG12, the positive CRMs. *G*, the activities of MG1–12 CRMs in the retina *ex vivo* were indicated by AP (Alkaline phosphatase) signals. The reporter plasmids containing each CRM were electroporated into P0 mouse retinas together with the electroporation efficiency control plasmid pCAG-mCherry. mCherry: electroporation efficiency control. Dark AP signals covered mCherry signals when CRM activity was strong. N ≥ 4. Scale bar: 50 μm. CRM, *cis*-regulatory module; MG, Müller glial cell; TFBS, transcription factor binding site.

Chromatin accessibility data alone cannot accurately annotate active CRMs. Many of the chromatin “open” CRMs are not active and fail to drive gene expression *in vivo* (19). To improve the efficiency of identifying active CRMs, we conducted a pilot study in which we tested the activities of chromatin accessible putative CRMs in the retina and compared the common features of active CRMs with those of inactive CRMs. We focused on retinal Müller glial cells (MG) because MG-enriched genes and a chromatin accessibility map of MG can be readily obtained from published single-cell RNA-seq and ATAC-seq datasets (17, 18). In addition, MGs play essential roles in maintaining the homeostatic environment of the retina (20). Recent studies further showed that MG can be reprogrammed into neurons *in vivo*, offering a promising treatment for retinal degenerative diseases (18, 21, 22). Therefore, identification of MG-specific CRMs and AAVs could be useful for studying MG under normal and disease conditions. We collected 19 chromatin accessible regions flanking five well-known MG-enriched genes, including *Rlbp1*, *Clu*, *Glul*, *Aqp4*, and *Ckdn1b* (23), based on published ATAC-seq from purified mouse MG (Fig. 1*A*). These putative CRMs were individually cloned into Stagia3 reporter plasmid, which expresses alkaline phosphatase (AP) when active CRM is inserted in front of the minimal TATA promoter (24, 25). The plasmids harboring different putative CRMs were electroporated into mouse retinas *ex vivo* at postnatal day 1 (P1). Electroporation mainly delivers plasmid DNA into mitotic retinal progenitor cells as nuclear envelop breakdown is necessary for the plasmids to enter the nuclei (26). P1 was chosen because retinal progenitor cells can differentiate into MG at this stage. The activities of CRMs were determined by AP staining 5 days later when retinal cell fate specification is largely finished. Four out of the 19 putative CRMs showed strong activity in the retina (Fig. 1*B*). We compared the sequences of active CRMs with inactive CRMs and tried to extract features correlating with CRM activity. Sequence conservation, ATAC-seq/DNase-seq peak height, and the number and density of total TFBSs within the CRM did not show any correlations with the activity of CRMs (Fig. S1, *A*–*E*). Interestingly, when we collected the TFBS matrices of MG-enriched TFs and scanned the 19 CRMs with these MG-TFBSs bioinformatically, the number of MG-TFBSs per 100 bp was positively correlated with the activity of CRMs (Fig. 1, *C* and *D*). This result suggests that prescreening of chromatin accessible putative CRMs based on the density of MG-TFBSs may significantly increase the efficiency of identifying active CRMs.

We further tested this prescreening strategy in retinal MG by increasing the scale of the screen (Fig. 1, *E*–*G*). We collected additional MG-enriched genes based on published single-cell RNA-seq data (16, 17) and literature (23), and selected putative CRMs flanking these MG-enriched genes based on chromatin accessibility revealed by ATAC-seq on MG (18) (Fig. 1*E*). The chromatin accessible regions flanking the genes specifically expressed in bipolar (BP), amacrine (AC), or photoreceptor (PR) cells were selected from the MG ATAC-seq data to serve as controls (Fig. 1*E*). We scanned a total of 149 putative CRMs with MG-TFBS matrices bioinformatically. The number of MG-TFBSs detected per 100 bp was presented in a two-way plot for each CRM (Fig. 1*F*). We randomly selected putative CRMs in quadrant A1–A3 or fell on the threshold lines of the quadrants as shown in Figure 1*F* and tested their activities in the retina *ex vivo*. All the active CRMs were in quadrant A2, which contains putative CRMs with higher density of MG-TFBSs (Fig. 1, *F* and *G*). When electroporated into mouse retinas *in vivo*, MG1, MG3, MG6, MG10, and MG12 showed robust and specific activities in MG (Fig. S2, *A*–*F*). Interestingly, the active CRMs were not only found flanking MG-enriched genes, but also near bipolar or amacrine-enriched genes (Fig. S1*F*). No putative CRMs flanking photoreceptor-enriched genes were found to have high density of MG-TFBSs and tested positive in the retina. Overall, these results suggest that the density of MG-TFBSs can be used as a predictor of CRM activity. To further support this conclusion, we generated a synthetic CRM containing TFBSs of 13 MG-specific TFs and found that it can drive MG-specific expression in the retina *ex vivo* (Fig. S2*G*).

### Integration of a shortened MG1 CRM into AAVs allows targeting of retinal Müller glial cells and brain astrocytes

**Figure 2:**
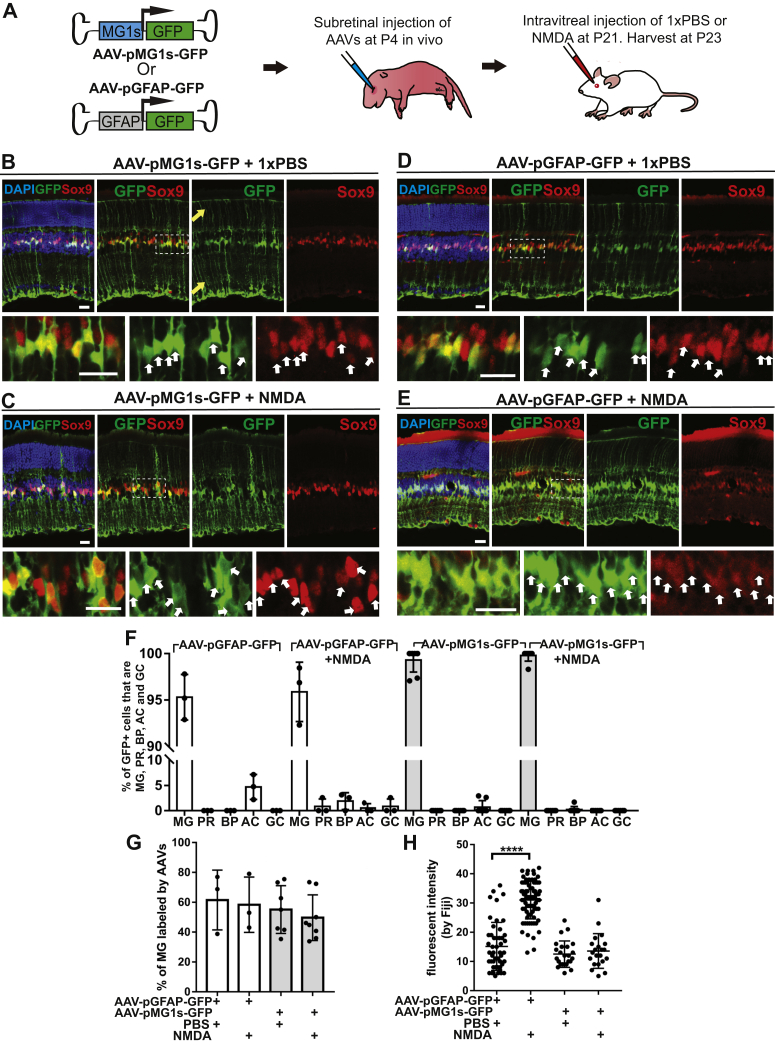
**The MG1s CRM in AAVs can target retinal MG and does not respond to retinal acute injury induced by NMDA.***A*, experimental design. WT mouse retinas were transduced by AAV-pMG1s-GFP or AAV-pGFAP-GFP with AAV8 capsid (10^12^ gc/ml) *via* subretinal injection at P4 to reach the highest transduction efficiency. At P21, 1xPBS (control) or NMDA (neurotoxin that can induce acute retinal injury) was injected into the same eye intravitreally. The retinas were harvested at P23. *B* and *C*, WT mouse retinas transduced by AAV-pMG1s-GFP (AAV8 capsid) with (*C*) or without (*B*) NMDA induced acute retinal injury. Higher magnification view of the highlighted region (*dotted box*) was shown for panel *B*–*E*. *Red*, Sox9 antibody staining labeled the nuclei/cell bodies of MG in inner nuclear layer (INL). *Green*, GFP signals were not amplified by immunostaining. *White arrow*, GFP+ MG. *Yellow arrow* (Panel *B*), the processes of MG. Scale bar: 20 μm. *D* and *E*, WT mouse retinas transduced by AAV-pGFAP-GFP (AAV8 capsid) with (*E*) or without (*D*) NMDA induced acute retinal injury. Scale bar: 20 μm. *F*, the percentage of AAV transduced cells that were MG, PR (photoreceptor), BP (bipolar cells), AC (amacrine cells) and GC (ganglion cells). N ≥ 3 mice. *G*, the percentage of MG that were labeled by AAV-pMG1s-GFP or AAV-pGFAP-GFP with or without NMDA induced injury. N ≥ 3 mice. *H*, the activities of the GFAP promoter and MG1s CRM under WT and injury conditions, indicated by the fluorescent intensity of GFP. PBS: 1xPBS control injection. Each dot represents the average GFP levels on each retina section slice. Two-tailed student *t* test. ∗∗∗∗*p* < 0.0001. Mean ± SD. N = 4 mice. AAV, adeno-associated virus; CRM, *cis*-regulatory module; MG, Müller glial cells.

We tried to integrate the identified MG CRMs into AAVs and test their activity and specificity in the mouse retina *in vivo*. We started with the MG1 CRM as it showed great specificity and efficiency when tested in reporter plasmids (Fig. S2*B*). AAV has a packaging limit of ∼4.7 kb. Shorter CRMs are favored as they can carry larger cargo genes. We first examined whether we could find the minimal element within the MG1 CRM (552 bp) that keeps the specificity and activity. We tested a shortened MG1 CRM (MG1s, 93 bp) containing two MG-TFBSs with the highest JASPAR scores. The higher the score, the higher the similarity between the sequence and TFBS matrix. When cloned into the reporter plasmid Stagia3, the MG1s CRM can drive the expression of *GFP* and *Cre*^*ER*^ specifically in MG *in vivo* (Fig. S2*H*). We then integrated MG1s into AAVs and tested its activity and specificity in the mouse retina *in vivo* (Fig. 2*A*). The MG1s CRM showed great specificity (∼99%) and efficiency (∼60%) in driving GFP expression in MG when integrated into AAVs (Fig. 2, *B*, *C*, *F* and *G*). In addition, we used Ai9 Cre-reporter mouse line, which turns on tdTomato expression upon Cre-mediated recombination, to examine whether MG1s can drive Cre^ER^ expression specifically in MG. The AAV-pMG1s-Cre^ER^ virus can turn on tdTomato expression specifically in MG in the presence of tamoxifen (50 mg/kg, intraperitoneal injection), demonstrating that it is a useful tool for manipulating MG *in vivo* (Fig. S3).

A few minipromoters that can drive MG-specific expression in AAVs in the retina have been reported previously (8, 21, 27, 28). The most widely used is GfabC1D (referred to as “GFAP promoter” in this manuscript), which was designed using DNA regions upstream of the *Gfap* gene (29). However, it is noteworthy that MG upregulate *Gfap* expression, a hallmark of gliosis, in response to injury and diseases (30, 31, 32, 33, 34, 35). We observed increased activity of the GFAP promoter compared to uninjured controls when we transduced the retina with AAV-pGFAP-GFP and acutely injured the retina with the neurotoxin NMDA (Fig. 2, *D*, *E* and *H*). The GFAP promoter may therefore lead to uncontrolled expression in disease models. Notably, the MG1s CRM did not respond to NMDA-induced injury (Fig. 2, *B*, *C* and *H*). The MG1s-containing AAV can be useful for targeting and studying MG under pathological conditions. Other reported minipromoters that were used in AAVs to limit the expression in MG were either longer than 2 kb or with relative low labeling efficiency. This limited their usage, especially for carrying large genes such as *Cas9* (27). The small size of the MG1s CRM (93 bp) is therefore advantageous. We summarized the specificity and efficiency of AAV-pMG1s-GFP/Cre/Cre^ER^ in targeting MG with different capsids and injection routes in Fig. S4.

**Figure 3:**
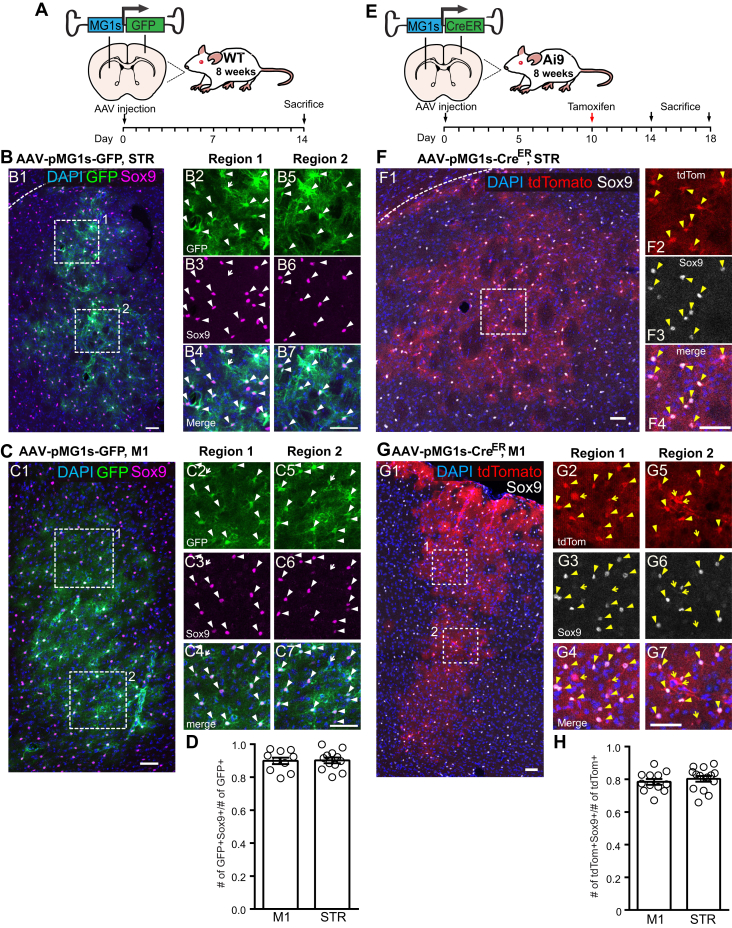
**MG1s CRM in AAVs can target astrocytes in adult brain.***A*, the schematics of AAV-pMG1s-GFP (AAV8 capsid) injection and experimental timeline. *B* and *C*, the striatum (STR, *B*) and motor cortex (M1, *C*) transduced with AAV-pMG1s-GFP in adult WT mice. *Magenta*, Sox9 antibody staining labeled the nuclei of astrocytes. B2-7, high magnification view of the highlighted regions in B1. C2–7, high magnification view of the highlighted regions in C1. *Filled arrow*, GFP+ Sox9+; *Open arrow*, GFP+ Sox9−. Scale bar: 100 μm. *D*, quantification of the labeling specificity of AAV-pMG1s-GFP in M1 and STR. n = 3 animals. *E*, the schematics of AAV-pMG1s-CreER (AAV8 capsid) injection and experimental timeline. Note: four mice were injected on Day 0, and two mice were sacrificed on day 14 and 18. Two groups showed very similar expressions, we combined them in the following analyses. *F* and *G*, the striatum (STR, *F*) and motor cortex (M1, *G*) transduced with AAV-pMG1s-CreER in adult Ai9 homozygous mice. *White*, Sox9 antibody staining labeled the nuclei of astrocytes. F2–4, high magnification view of the highlighted region in F1. G2–7, high magnification view of the highlighted regions in G1. *Filled arrow*, tdTomato+ Sox9+; *Open arrow*, tdTomato+ Sox9−. Scale bar: 100 μm. *H*, quantification of the labeling specificity of AAV-pMG1s-CreER in M1 and STR. N = 4 mice. AAV, adeno-associated virus; CRM, *cis*-regulatory module; MG, Müller glial cells.

In addition, as retinal MGs share a similar gene expression profile with brain astrocytes (23), we also tested the specificity of AAV-pMG1s-GFP/Cre^ER^ in the brain. When injected into the motor cortex (M1) or striatum (STR) of 8-week-old adult wild-type mice, the AAV-pMG1s-GFP virus can label astrocytes with great specificity (>90%) (Fig. 3, *A*–*D*). Similarly, the AAV-pMG1s-Cre^ER^ virus can specifically activate Cre-reporter line Ai9 and turn on tdTomato expression in astrocytes in the striatum and motor cortex (Fig. 3, *E*–*H*). These AAVs can be useful tools for manipulating astrocytes in the brain.

### Synthetic “stitching” CRMs can render cell type specificity in AAVs in the retina

For many cell types or subtypes in the retina and brain, the cell-type-specific TF/TFBS profile and chromatin accessibility data may not be readily available. To develop AAVs targeting these cell types, we investigated whether generating a synthetic “CRM” would render cell type specificity. CRMs are highly sensitive to DNaseI. The ENCODE project has published the DNase-seq data on a variety of tissues including the whole retina and brain (11, 12). Instead of testing individual DNaseI hypersensitive regions, we tested whether stitching the DNaseI hypersensitive regions flanking a cell-type-specific gene together can render activity and specificity in AAVs.

**Figure 4:**
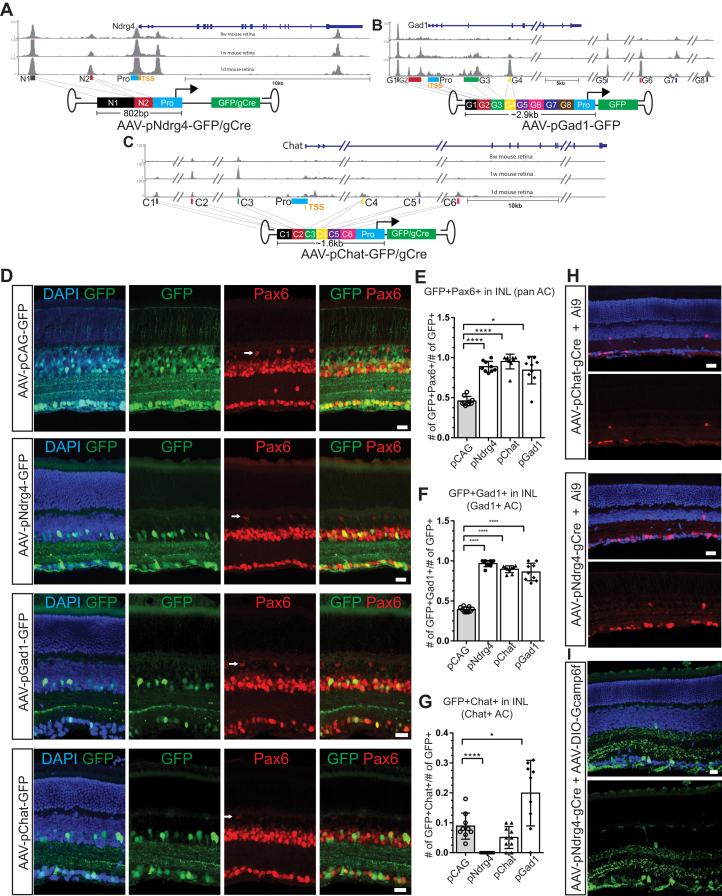
**Synthetic CRMs in AAVs can render cell type-specificity in retinal amacrine cells.***A*–*C*, the schematics of the synthetic CRMs. *D*, WT mouse retinas transduced with AAV-pCAG-GFP (Control), AAV-pNdrg4-GFP, AAV-pGad1-GFP, and AAV-pChat-GFP with AAV7m8 capsid (*via* intravitreal injection). Pax6: amacrine cell marker in the inner nuclear layer (INL). *White arrow*, horizontal cells that were also labeled by Pax6. Scale bar: 20 μm. *E*, the percentage of GFP+ cells that were Pax6+ amacrine cells in the inner nuclear layer (INL). *F*, the percentage of GFP+ cells that were Gad1+ in INL. *G*, the percentage of GFP+ cells that were Chat+ in INL. pCAG: AAV-pCAG-GFP; pNdrg4: AAV-pNdrg4-GFP; pChat: AAV-pChat-GFP; pGad1: AAV-pGad1-GFP. Two tailed student *t* test. ∗*p* < 0.05; ∗∗∗*p* < 0.001; ∗∗∗∗*p* < 0.0001. Mean ± SD. N ≥ 4 mice. *H*, Ai9 homozygous mouse retinas transduced with AAV-pChat/Gad1-gCre (AAV7m8 capsid) at P4 and harvested at P30. *I*, WT mouse retinas transduced with AAV-pNdrg4-gCre and AAV-DIO-Gcamp6f (AAV7m8 capsid) at P4 and harvested at P30. Scale bar: 20 μm. AAV, adeno-associated virus; CRM, *cis*-regulatory module.

We first used this strategy to generate AAVs targeting retinal amacrine cells (AC), which are essential interneurons of the visual circuits (36) and with limited molecular tools for labeling and manipulation. We tried to prescreen for candidate AC- or AC-subtype-specific CRMs using AC-enriched TFBSs but failed in that we cannot collect enough AC or AC-subtype-enriched TFs based on the published single-cell RNA-seq data (17). We therefore decided to stitch the DNaseI hypersensitive regions flanking AC-enriched genes and examine whether this strategy can lead to AAVs targeting AC. We focused on three genes that are highly enriched in all AC, or subtypes of AC, including *Ndrg4* (N-myc downstream regulated gene 4), *Chat* (Choline O-sacetyltransferase), and *Gad1* (Glutamate decarboxylase 1) (Fig. 4, *A*–*C*). Based on the published single cell RNA-seq data and the available literature, Ndrg4 is expressed in all types of retinal AC and some ganglion cells (17) (Fig. S5). A 6.2 kb promoter of the *Ndrg4* gene can drive expression specifically in AC *via in vivo* electroporation (26). Gad1, the enzyme for producing GABA, is a well-known marker for GABAergic AC in the retina. CHAT expressing AC, which is essential for generating direction-selective responses to motion (37), is a subtype of GABAergic AC. For each of these three genes, we collected DNaseI hypersensitive regions flanking the gene in mouse retina at P1, P7, and 8 weeks of age based on the DNase-seq datasets published by the ENCODE project. We prioritized DNaseI hypersensitive regions detected specifically in the retina and rejected those that were simultaneously present in the brain and retina (Fig. 4, *A*–*C*). The synthetic CRMs were generated by stitching these DNaseI hypersensitive regions together and cloned into AAV backbones. A modified AAV2 capsid (AAV7m8), previously shown to transduce inner retinal cell types *via* intravitreal injection (38), was used in combination with engineered AAV backbones.

We injected AAV-pNdrg4-GFP, AAV-pChat-GFP, or AAV-pGad1-GFP into the mouse eyes *in vivo* at P4 *via* intravitreal injection (Fig. 4). The P4 stage was chosen in that AAV infection efficiency is much higher at P4 due to relatively small eyeball size at this stage. The retinas were harvest at P21 for histology. The specificity of these AAVs was compared with the control AAV, in which a ubiquitous CAG promoter drives GFP expression. The Ndrg4, Chat, and Gad1 AAVs directed GFP expression specifically in AC in the inner nuclear layer (INL) compared with AAV-pCAG-GFP, as shown by colabeling with a pan-amacrine marker Pax6 (Fig. 4, *D*–*G*). Interestingly, all three AAVs specifically targeted Gad1+ GABAergic AC, despite the expression of Ndrg4 in all types of amacrine cells (Fig. S5*B*). In ganglion cell layer (GCL), Gad1 AAVs were able to target displaced AC, whereas Ndrg4 and Chat AAVs labeled displaced AC and a percentage of ganglion cells (Fig. S6). The Ndrg4 and Chat AAVs did not label any CHAT positive cells in either INL or GCL, while the Gad1 AAVs labeled fewer CHAT positive AC than the AAV-pCAG-GFP control (Figs. 4*G* and S6).

We also investigated whether the AC-targeting stitching CRMs allow cell-type-specific genetic manipulation when integrated into AAVs. When the *Cre* gene was inserted into the AC-targeting AAVs, Cre-reporter genes were activated nonspecifically, possibly due to minimal stochastic activity of synthetic CRMs and high efficiency of Cre recombinase (Fig. S7, *A* and *B*). To achieve greater specificity, we generated a modified gCre recombinase by fusing Cre with human geminin protein. Geminin, a key regulator of the cell cycle, is degraded in the G1/G0 phase of the cell cycle (39). Fusing Cre recombinase with geminin successfully desensitized the Cre activity and reduced nonspecific recombination in postmitotic cells in the retina and brain (Fig. S7). We used the AC-targeting stitching CRMs to drive gCre expression in AAVs, and injected these AAVs into the eyes of Cre-reporter Ai9 mice. pGad1-gCre is too large to fit into the AAV backbone (>4.7 kb). AAV-pChat-gCre and AAV-pNdrg4-gCre activated expression of the tdTomato reporter gene sparsely in AC and some ganglion cells (Fig. 4*H*). These sparse-labeling AAV tools can be useful for studying the function and circuits of AC at single cell levels. For instance, genetically encoded calcium indicators (GCaMPs) have been widely used to monitor neuronal activity. Coinjection of AAV-pNdrg4-gCre and AAV-DIO-CAG-GCaMP6f (40) (AAV expressing GCaMP6f in a Cre-dependent manner) into wild-type mouse eyes allowed delivery of the GCaMP6f indicator into single AC (Fig. 4*I*).

Taken together, these results demonstrated that AAVs with synthetic stitching CRMs from AC-enriched genes can label Gad1+ GABAergic AC and provide opportunities for manipulating individual AC in the mouse retina *in vivo*.

### AAVs with synthetic CRMs can target parvalbumin (PV)-positive neurons in the brain

PV-positive GABAergic interneuron is one of the major interneuron types in the brain and play important roles in microcircuit regulation. A few AAVs targeting cortical PV+ interneurons have been developed by integrating putative CRMs into AAVs (9, 10). These CRMs were selected based on bulk/scATAC-seq of cortical regions. However, their specificity and efficiency of labeling subcortical PV+ cells were low. The current stitching approach utilizes the DNase-seq data from whole brain (ENCODE project), including both cortical and subcortical regions. This strategy may generate AAVs that can target PV+ interneurons in multiple brain regions.

**Figure 5:**
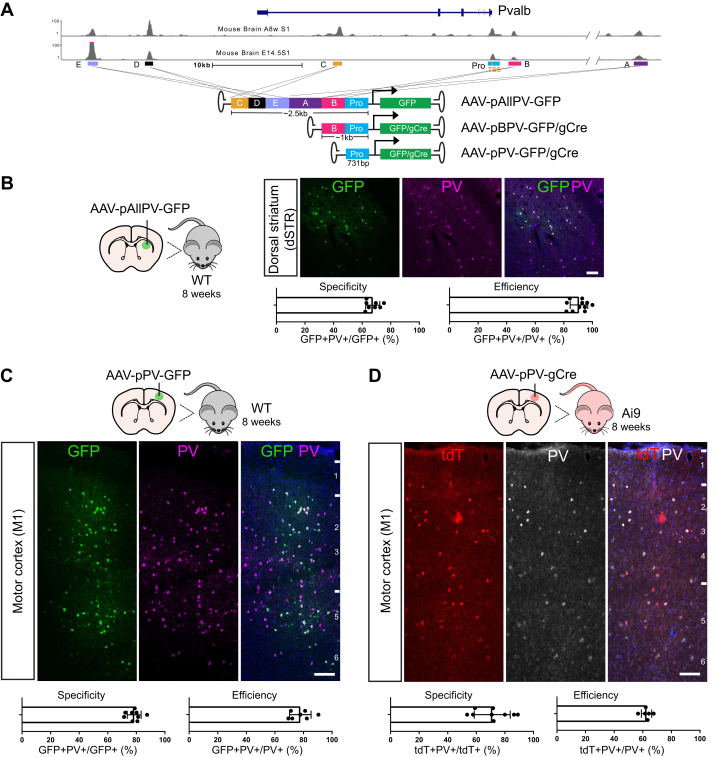
**AAVs with synthetic CRMs can target PV+ neurons in the brain.***A*, the schematics of the PV synthetic CRMs. *B*–*D*, representative images of infected brain regions and quantification of the labeling specificity and efficiency of AAV-pAllPV-GFP (*B*), AAV-pPV-GFP (*C*), and AAV-pPV-gCre (*D*). For each panel, *top*, experimental design. *Middle*, representative images showing AAV labeled cells (GFP or tdTomato signals) and PV antibody staining. Scale bar: 100 μm. *Bottom*, quantification of the labeling specificity and efficiency. Mean ± SD. N ≥ 4 mice. AAV, adeno-associated virus; CRM, *cis*-regulatory module; PV, parvalbumin.

We identified five putative CRMs (A–E) near the *Pvalb* gene by investigating the DNaseI hypersensitive peaks of mouse whole brain published by the ENCODE project (Fig. 5*A*). These putative CRMs were stitched together, placed in front of the PV promoter, and inserted into AAV backbones (AAV-pAllPV-GFP). AAV-pPV-GFP (promoter only, determined based on DNaseI hypersensitivity) and AAV-pBPV-GFP (promoter with CRM B) were also generated to serve as controls. These AAVs with AAV8 capsid were injected into two cortical regions of adult mouse brain, motor cortex (M1) and visual cortex (V1), and one subcortical region, striatum. All three AAVs led to robust GFP expression in the infected regions. Interestingly, we found that the specificity of these AAVs varied across regions (Table S1). The AAV-pAllPV-GFP virus had good specificity (∼68%) in labeling PV+ neurons in striatum, but the specificity dropped to 35% in motor cortex (Figs. 5*B* and S8). The AAV-pPV-GFP virus showed a higher specificity in motor cortex (∼79%) than in striatum (∼44%) (Figs. 5*C* and S8). The AAV-pBPV-GFP virus showed the highest specificity in visual cortex (∼88%) but relatively low specificity in both motor cortex and striatum (∼40%) (Fig. S8). In addition, we tested whether the synthetic PV CRMs allowed genetic manipulation of PV+ interneurons *via* Cre recombinase. As we observed in the retina, Cre expression driven by the PV CRMs led to nonspecific labeling in Ai9 reporter mice (Fig. S7, *C* and *D*). We therefore placed gCre into the PV AAVs. AAV-pPV-gCre and AAV-pBPV-gCre viruses with AAV8 capsid sparsely labeled PV+ interneurons in M1 and striatum of Ai9 mice with decent specificity (Figs. 5*D* and S8; Table S1) and could be used to manipulate PV+ interneurons *in vivo*. AAV-pAllPV-gCre cannot be generated because the size of pAllPV-gCre exceeds the packaging limit of AAVs (>4.7 kb).

## Discussion

We explored strategies that can increase the efficiency of identifying CRMs for AAV-based cell-type-specific labeling and manipulation. We showed that prescreening of chromatin accessible putative CRMs based on the density of cell-type-enriched TFBSs can significantly increase the efficiency of identifying active CRMs. In addition, generation of synthetic CRMs by stitching chromatin accessible regions flanking cell-type-enriched genes can straightforwardly render cell type specificity in AAVs in many cases. Both strategies utilize available large-scale datasets in the literature and allow generating of AAVs that can target specific CNS cell types without conducting comprehensive screens and large-scale sequencing analyses.

Previous studies have screened for CRMs or synthetic promoters that can target specific cell types when integrated into AAVs in the retina and brain. The screen reported by Jüttner *et al.* (8) relied on the generation and testing of a large amount of AAVs harboring different synthetic promoters, which is time-consuming and relatively inefficient. Graybuck *et al.* and Mich *et al.* performed single-cell ATAC-seq of mouse brain tissues and utilized the data to nominate CRMs for AAVs (10, 41). This strategy improved the efficiency of identifying active CRMs, but it highly relies on the success of single-cell ATAC-seq analyses, which could be relatively complicated to perform, especially for low-abundant cell types. Certain chromatin accessible regions in the genome also may not be revealed by single-cell ATAC-seq because of the depth of single-cell sequencing.

The major strength of the prescreening strategy is that it they can increase the efficiency of identifying cell-type-specific CRMs using available genomic data. The results of our analysis suggest that putative CRMs containing >1 (JASPAR score > 11) or >1.7 (JASPAR score > 10) MG-TFBSs per 100 bp are likely active CRMs in MG. (Fig. 1*F*). The major strength of the stitching strategy is that it is easy to apply and highly practical, especially for cell types with limited information on chromatin accessibility.

Both strategies have limitations and can be improved. We formulated and tested the prescreening strategy using retinal MG as a model system. It would be important to apply this strategy to other retinal or brain cell types as more cell-type-specific ATAC-seq, scATAC-seq, and RNA-seq data become available. In addition, we weighted all the MG-enriched TFBSs equally in this study because it is difficult to predict the power and activity of TFs in retinal cells *in vivo*. Weighting TFBSs differentially based on their affinity and activity is probably necessary for better prediction. The stitching strategy can restrict gene expression to desired neuronal cell types and has comparable efficiency to other published strategies (Table S1). But AAVs generated by this strategy may fail to fully recapitulate the endogenous gene expression patterns. For example, the stitched CRMs designed based on the chromatin open regions flanking the *Ndrg4*, *Gad1*, and *Chat* genes all favored the labeling of Gad1+Chat-cells in AAVs and failed to mark any Chat+ cells in the retina. This is not due to the accessibility of the AAV7m8 capsid that we used, because control AAV-pCAG-GFP with AAV7m8 capsid can label Chat+ cells. One possibility, which needs to be further investigated, is that chromatin accessible silencer CRMs may be included in these stitched CRMs.

Apart from identifying active CRMs for AAVs, the prescreening and stitching strategies also allow for revealing of unknown biology regarding gene regulation. Using the prescreening strategy, we found that the putative CRMs with high density of MG-enriched TFBSs were not only found near MG-enriched genes, but also present near bipolar and amacrine-enriched genes. For example, the MG1 CRM with high density of MG-enriched TFBSs resides in the intron of the *Trmp1* gene, which is well known to be expressed in retinal bipolar cells but not in MG. Therefore, some bipolar or amacrine-enriched genes cannot be transcribed in MG even though they are near the CRMs that can be activated in MG. Additional mechanisms may be needed to repress the expression of bipolar or amacrine-enriched genes in MG. In the brain, we tested and compared multiple chromatin accessible putative CRMs flanking the *Pvalb* gene by stitching them together in AAVs. PV promoter and CRM B showed good labeling of cortical PV+ neurons (M1 and V1). Adding CRM A, C, D, and E regions led to better targeting of subcortical PV+ neurons, indicating that there may be putative striatum-specific CRMs among these CRMs (Fig. 4).

In addition, we introduced a new tool to the field. The previously reported cell-type-specific synthetic promoters or CRMs often led to nonspecific labeling when driving the expression of Cre or destabilized Cre recombinase (Fig. S8). The gCre protein generated here can significantly reduce the sensitivity of Cre and increase the gene manipulation specificity mediated by Cre recombinase in AAVs.

In summary, we tested two straightforward strategies for increasing the efficiency of identifying cell-type-specific CRMs for AAVs and generated useful AAV tools for targeting retinal MG and AC and brain astrocytes and PV+ neurons. Application of these strategies in other CNS cell types could significantly improve the efficiency of generating AAV tools targeting specific cell types.

## Experimental procedures

### Animals

Wild-type mouse neonates were obtained from time pregnant CD1 mice (Charles River Laboratories, #022). C57BL/6J (Stock No: 000664) and Ai9 (Stock No: 007909) mice were purchased from the Jackson Lab. All animal studies were approved by the Administrative Panel on Laboratory Animal Care (APLAC) at Stanford University.

### Plasmid construction

The CAG-mCherry plasmid was from Matsuda and Cepko (42). The Stagia3 vector was from Billings *et al.* (24). The AAV-GFAP-GFP plasmid was a gift from Dr Wenjun Xiong (City College of Hong Kong). All other plasmids were constructed by restrictive enzyme-based cloning.

#### CRM reporter plasmids (Fig. 1)

The putative CRMs were amplified from the mouse genome by PCR and cloned into the Stagia3 vector. The sequences of putative CRMs and promoters were listed in Supplementary File 1.

#### AAV plasmids (Figure 2, Figure 3, Figure 4, Figure 5)

The AAV-pMG1s-GFP plasmid was generated by replacing the GFAP promoter of the AAV-pGFAP-GFP plasmid with MG1s. The AAV-pMG1s-CreER and AAV-pGFAP-CreER plasmid were generated by replacing the GFP of the AAV-pMG1s-GFP and AAV-pGFAP-GFP plasmids with CreER (Addgene #14797) (26). The Ndrg4, Gad1, and Chat synthetic CRMs were synthesized by IDT and cloned into AAV vectors. gCre was generated by fusing the human Geminin protein (amino acid 1–110) to the C terminus of Cre recombinase. AAV-Ndrg4/Chat-gCre was generated by replacing the GFP of the AAV-Ndrg4/Chat-GFP plasmid with gCre. PV CRMs were PCR amplified from mouse genomic DNA, stitched by Gibson assembly (NEB, Cat.No. E2611), and cloned into AAV backbones. The sequences of synthetic CRMs and gCre were listed in Supplementary File 1.

### AAV production and delivery

Recombinant AAV8, AAV7m8, and AAVshH10 viruses were produced as previously described (43). The AAV8 and AAV7m8 capsid plasmids were gifts from Dr Wenjun Xiong and Dr Connie Cepko (Harvard Medical School). The shH10 capsid was a gift from Drs John Flannery & David Schaffer (Addgene plasmid # 64867). SDS-PAGE gels were used to determine the titer of AAVs.

AAV subretinal injection was performed on P3-P4 neonatal mice as previously described (25, 42). Approximately 0.5 to 1 μl AAV8 (10^12^–10^13^ gc/ml) was delivered into the subretinal space. AAV intravitreal injection was performed on P4 mice using a pulled glass needle controlled by Femtojet (Eppendorf). Approximately 1 to 2 μl AAV7m8 or AAVshH10 (10^12^–10^13^ gc/ml) was delivered into the intravitreal space.

Viruses were delivered to adult brains by stereotaxic injection. Briefly, mice were anesthetized by 1.5 to 2.0% isoflurane and placed on a stereotaxic frame (Kopf instrument). A glass needle was prepared by micropipette puller, filled with virus solution, and fixed on the micromanipulator. Then, the needle was moved to the desired position over a predrilled hole in the skull and slowly lowered to the target depth. In total, 0.5 μl of virus solution was injected at an infusion rate of 50 to 100 nl/min. Following virus injection, the scalp was sutured, and mice were returned to their home cages for 2 weeks before tissue harvesting. To locate specific areas, the following coordinates were used: M1, AP: 1 mm, ML: ±1.5 mm from bregma, DV: −1.0 mm from brain surface; STR, AP: 1.2 mm, ML: ±2 mm from bregma, DV: −2.2 mm from brain surface; V1, AP: −2.8 mm, ML: ±2.2 mm, DV: −1.5 mm from bregma.

### *Ex vivo* and *in vivo* plasmid electroporation into the retina

*Ex vivo* and *in vivo* retina electroporations were carried out as previously described (25, 26, 42). For *ex vivo* electroporation, five pulses of 25 V, 50 ms each, and 950 ms intervals were applied to dissected retinas. For *in vivo* electroporation, five pulses of 80 V, 50 ms each, and 950 ms intervals were applied to neonatal mouse pups. All *ex vivo* and *in vivo* electroporation experiments were repeated with at least three biological replicates. Plasmids were electroporated with a concentration of 500 ng/μl to 1 μg/μl per plasmid.

### Histology and immunohistochemistry

Dissected mouse eyes were fixed in 4% paraformaldehyde (PFA) in PBS (pH 7.4) for 2 h at room temperature. Retinas then were dissected and equilibrated at room temperature in a series of sucrose solutions (5% sucrose in PBS, 5 min; 15% sucrose in PBS, 15 min; 30% sucrose in PBS, 1 h; 1:1 mixed solution of OCT) and 30% sucrose in PBS, 4 °C, overnight, frozen and stored at −80 °C. A Leica CM3050S cryostat (Leica Microsystems) was used to prepared 20 μm cryosections. Retinal cryo-sections were washed in PBS briefly, incubated in 0.2% Triton X-100 (Sigma-Aldrich), PBS for 20 min, and blocked for 30 min in blocking solution, which is 0.1% Triton, 1% BSA, and 10% Donkey Serum (Jackson ImmunoResearch Laboratories, Inc) in PBS. Slides were incubated with primary antibodies diluted in blocking solution in a humidified chamber at room temperature for 2 h or at 4 °C overnight. After washing in 0.1% Triton PBS three times, slides were incubated with secondary antibodies and DAPI (Sigma-Aldrich; D9542) for 30 min to 2 h, washed three times with 0.1% Triton PBS, and mounted in Fluoromount-G (Southern Biotechnology Associates).

To harvest the brains, mice were anesthetized with isoflurane and transcardially perfused with PBS followed by 4% PFA/PBS. Brains were collected, post-fixed in 4% PFA/PBS for 4 h, and dehydrated in 30% sucrose/PBS for 2 days at 4 °C. Brains were sectioned into 30 μm slices by a cryotome or cryostat (Leica Microsystems). Floating slices were blocked with PBS containing 10% Donkey Serum and 0.5% Triton X-100 at room temperature for 2 h. Then the slices were incubated with primary antibodies and second antibodies (as described above) and mounted in VECTASHIELD(R) Mounting Medium with DAPI (Vector Laboratories, H1500).

The following primary antibodies were used: chicken anti-GFP (Abcam, AB13970, 1:1000), mouse anti GAD67 (Gad1, Sigma, ZMS5406–25 μl, 1:500), rabbit anti CHAT (sigma, AB144P-200UL, 1:200), rabbit anti Sox9 (Abcam, AB185966, 1:500), rabbit anti Pax6 (ThermoFisher, 42-6600, 1:500), guinea pig anti RBPMS (PhosphoSolutions, 1832-RBPMS, 1:500), and mouse anti-parvalbumin (Swant, PV 235, 1:1000) antibodies. AP activity was detected by an AP detection kit (Sigma, SCR004).

### Imaging and analysis

All images of retinal and brain sections were acquired by a Zeiss LSM880 inverted confocal microscope. Retina explants were imaged by Leica M165 FC microscope. Images in Figures 2 and 4 were maximum projections of 5 to 10 μm tissues and were quantified by Fiji software. Images in Figures 3 and 5 were projections of 30 μm tissues.

### Collection of cell-type-enriched genes/TFs, putative CRMs, and TFBS density analyses

MG-enriched TFs (Fig. 1*B*) and MG, BP, AC, or PR-enriched genes (Fig. 1*E*) were collected based on available single-cell RNA-seq (scRNA-seq) data on adult mouse retinas (17) and published literature (23). The scRNA-seq gene expression matrix was downloaded from GSE63472, and the computational analysis was performed by using the SEURAT2 pipeline. The cells that contained less than 20% mitochondrial reads and more than 900 genes per cell were kept for downstream analyses, which included the standard functions NormalizeData, FindVariableGenes, ScaleData, RunPCA, FindClusters (resolution of 0.6), RunTSNE, and FindAllMarkers. The criteria for selecting cell-type-enriched genes and TFs were as follows (using MG-enriched genes/TFs as examples). First, we selected the genes that are significantly enriched in MG cluster in the scRNA-seq data. Specifically, the Seurat “FindAllMarkers” function with default parameters (object = pbmc, only.pos = FALSE, min.pct = 0.25, thresh.use = 0.25) was used to identify marker genes enriched in MG cluster. The Wilcoxon Rank Sum test was used to generate *p* values. The average expression level of each marker gene in MG cluster *versus* all other cell clusters was calculated as Log_2_(FC) (the log-ratio of fold change). We considered genes with Log_2_(FC) >2 and *p* value <0.05 as MG-enriched genes. Among these MG-enriched genes, we selected genes encoding transcription factors as MG-enriched TFs. The scripts for these analyses are available at: https://github.com/tanasa/the_scripts_analysis_scRNAseq.

To collect putative CRMs (Fig. 1), we downloaded the BigWig files for DNase-seq of mouse retina tissue from the ENCODE portal (https://doi.org/10.1093/nar/gkx1081) (https://www.encodeproject.org/) (12) and raw ATAC-seq data for MG from NCBI Sequence Read Archive (accession SRX2881310) (18). The raw ATAC-seq data were analyzed using the Galaxy platform (usagalaxy.org) following standard pipelines. Specifically, reads were aligned to mouse mm9 genome using Bowtie2 (44) and processed with MarkDuplicates to locate duplicate reads. MACS2 callpeak function was used to generate with narrow peaks. The Wig/BedGraph-to-bigWig function with default parameters was used to generate BigWig coverage tracks, which were visualized in UCSC genome browser. We selected putative CRMs (peaks in ATAC-seq or DNase-seq data) using the following criteria manually. (1) We scanned the regions flanking a gene of interest until the neighboring genes (UCSC genes, mouse mm9) in BigWig coverage tracks. If an ATAC peak overlapped with an exon, we excluded it from the selection. (2) We collected peaks with the tip signal values ≥20. The left or right boundary of the peak (putative CRM) was set when the signal value reached 20% of the tip signal value. All the sequences of the selected putative CRMs/peaks were listed in Supplementary File 2.

The TFBS matrices of MG-enriched TFs were obtained from JASPAR database (jasper.genereg.net) (45), and manually imported into JASPAR website. We used the “SCAN” function (Relative profile score threshold 80%) in JASPAR website to predict TFBSs within each putative CRM. The TFBSs with JASPAR score >10 or >11 were collected. If the start of a TFBS is within 5 bp from the start of another TFBS, we considered them as 1 “TFBS count.” The number of “TFBS count” was calculated for each putative CRM. The MG-TFBS per 100 bp (Fig. 1*F*) is “TFBS count” divided by the length of CRM (bp) ∗100. We wrote a python program to automatically count TFBSs and calculate the number of “TFBS count” per 100 bp (Supplementary File 2). If a CRM contains long tandem repeats (such as CTCCCT or ACACACACACACAC), we manually correct the data by skipping the repeated region.

## Data availability

All data are contained within the manuscript.

## Supporting information

This article contains supporting information.

## Conflict of interest

The authors declare that they have no conflicts of interest with the contents of this article.
