# Supplementary material for: Identification of cis-regulatory modules for adeno-associated virus-based cell-type-specific targeting in the retina and brain
Source: J Biol Chem. 2022 Feb 9;298(4):101674. doi: 10.1016/j.jbc.2022.101674 (PMC8980332; doi:10.1016/j.jbc.2022.101674)
Supplement: Supplemental Figures S1–S8 and Table S1 [file mmc3.pdf]

Identification of *cis*-regulatory modules for adeno-associated virus-based cell-type-specific targeting in the retina and brain

Supplementary materials:

1. Supplementary Figure 1-8.
2. Supplementary Table 1 (Table S1).

Fig S1

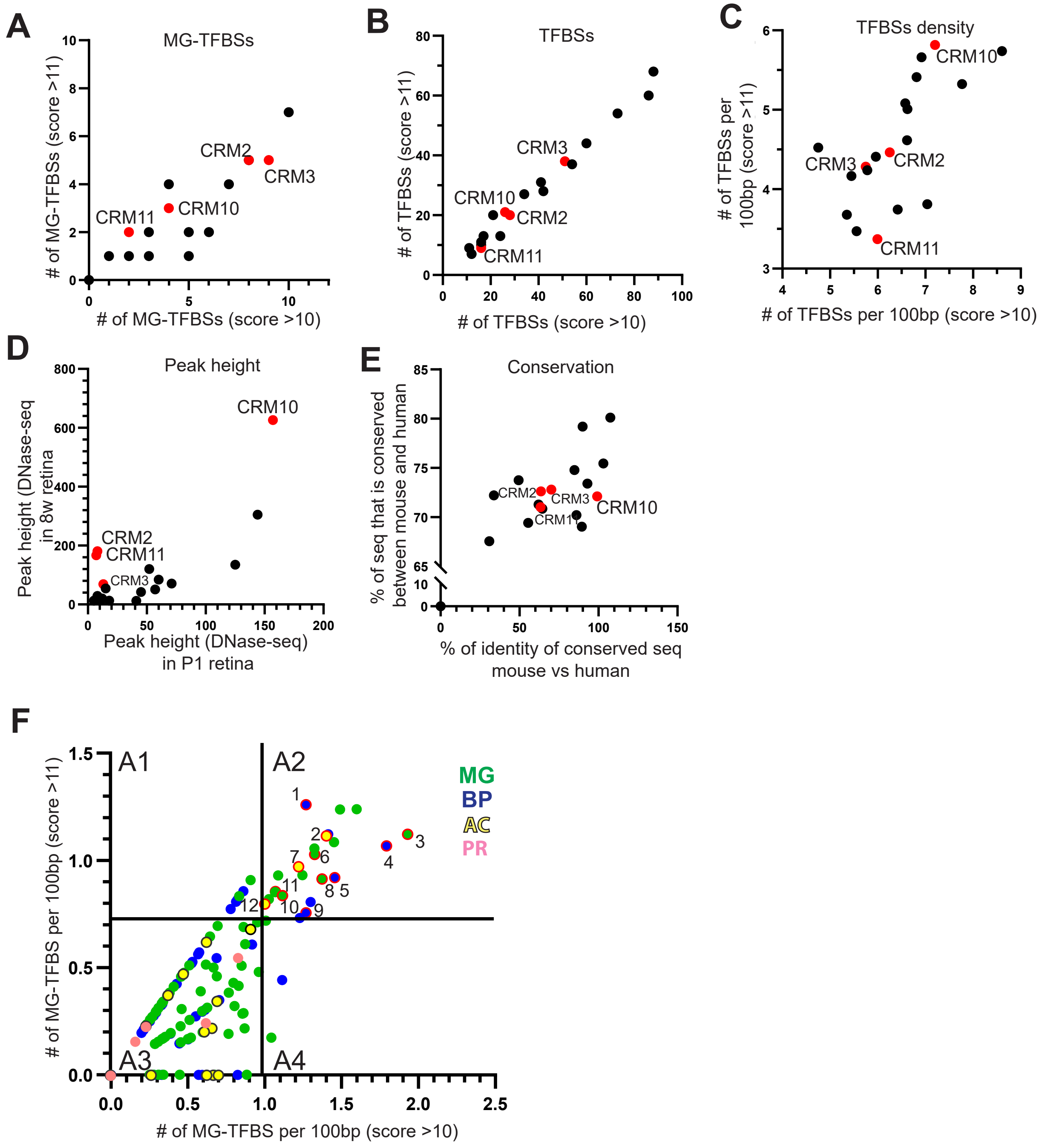
A
MG-TFBSs
# of MG-TFBSs (score >11)
# of MG-TFBSs (score >10)
CRM2
CRM3
CRM10
CRM11
B
TFBSs
# of TFBSs (score >11)
# of TFBSs (score >10)
CRM3
CRM10
CRM2
CRM11
C
TFBSs density
# of TFBSs per 100bp (score >11)
# of TFBSs per 100bp (score >10)
CRM10
CRM3
CRM2
CRM11
D
Peak height
Peak height (DNase-seq in 8w retina
Peak height (DNase-seq) in P1 retina
CRM10
CRM2
CRM11
CRM3
E
Conservation
% of seq that is conserved between mouse and human
% of identity of conserved seq mouse vs human
CRM2
CRM3
CRM10
CRM11
F
A1
A2
A3
A4
MG
BP
AC
PR
# of MG-TFBS per 100bp (score >11)
# of MG-TFBS per 100bp (score >10)

Fig. S1. The prescreening strategy for identifying cell-type-specific active CRMs using retinal Müller glial cell (MG) as a model system (associated with Fig 1).

(A-E) The total number of MG-TFBSs (TFBSs of MG-enriched TFs) (A), TFBSs (all available TFBSs) (B), TFBS density (the number of TFBSs per 100bp) (C), peak height (DNase-seq) (D), and sequence conservation (E) of CRM1-19 were plotted. Red: positive CRMs in the retina; Black: negative CRMs in the retina. For TFBSs and MG-TFBSs, x axis is the number of TFBSs or MG-TFBSs per 100bp with JASPAR relative score >=10. Y axis is the number of TFBSs or MG-TFBSs per 100bp with JASPAR relative score >11. Peak height: the DNaseI hypersensitive peaks revealed by DNase-seq (ENCODE). Conservation: sequence conservation between mouse and human (based on ECR browser). **(F)** The plot showed in Fig 1F. Here the putative CRMs flanking MG (green, Müller glia), BP (blue, bipolar cells), AC (yellow, amacrine cells) or PR (pink, photoreceptor cells)-enriched genes were highlighted based on cell types. Positive CRMs were highlighted by red outlines. A1-A4: four quadrants of the plot.

Fig S2

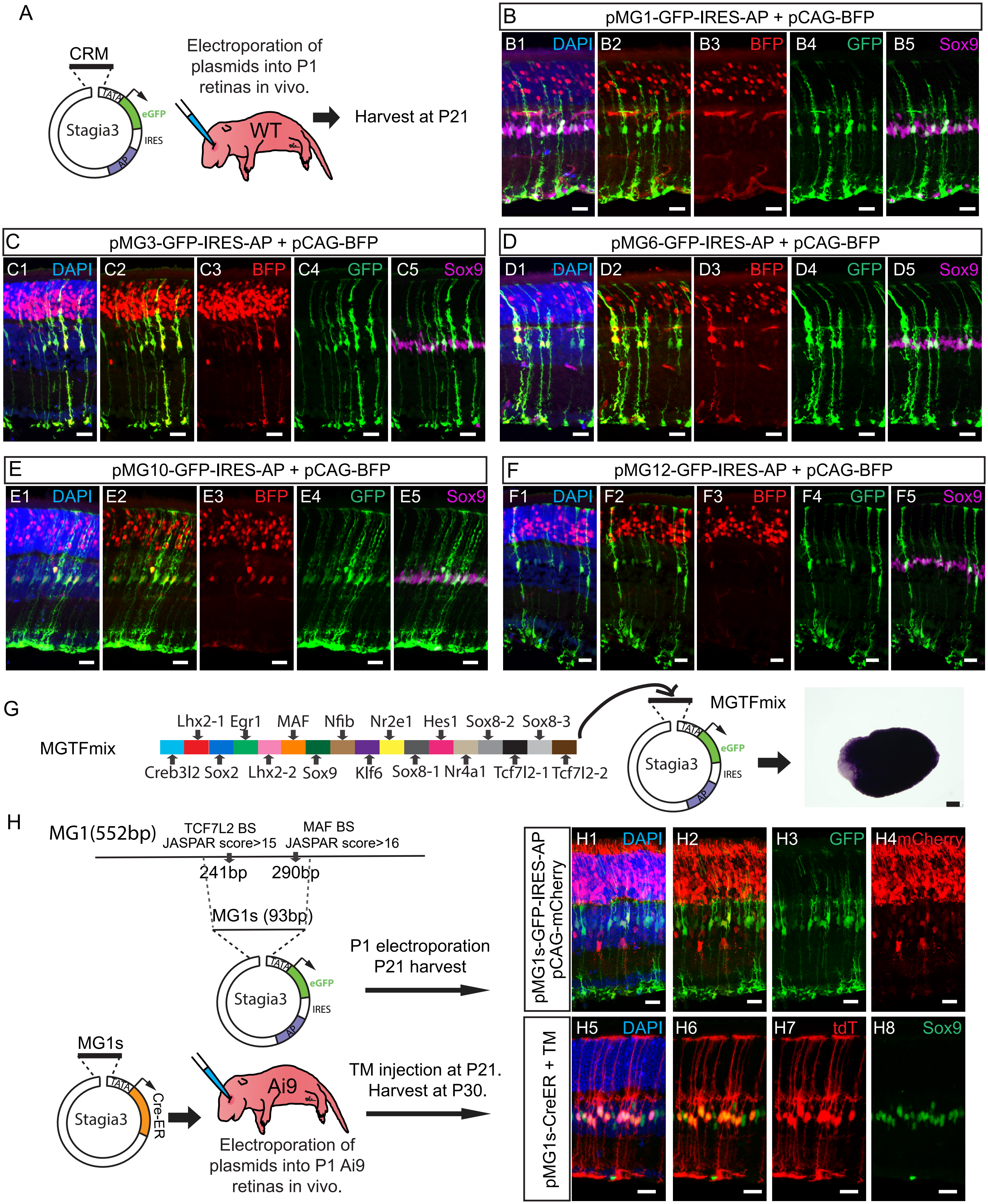
A
CRM
Stagia3
TATA
eGFP
IRES
AP
Electroporation of plasmids into P1 retinas in vivo.
WT
Harvest at P21
B
pMG1-GFP-IRES-AP + pCAG-BFP
B1 DAPI
B2
B3 BFP
B4 GFP
B5 Sox9
C
pMG3-GFP-IRES-AP + pCAG-BFP
C1 DAPI
C2
C3 BFP
C4 GFP
C5 Sox9
D
pMG6-GFP-IRES-AP + pCAG-BFP
D1 DAPI
D2
D3 BFP
D4 GFP
D5 Sox9
E
pMG10-GFP-IRES-AP + pCAG-BFP
E1 DAPI
E2
E3 BFP
E4 GFP
E5 Sox9
F
pMG12-GFP-IRES-AP + pCAG-BFP
F1 DAPI
F2
F3 BFP
F4 GFP
F5 Sox9
G
MGTFmix
Lhx2-1 Egr1 MAF Nfib Nr2e1 Hes1 Sox8-2 Sox8-3
Creb3l2 Sox2 Lhx2-2 Sox9 Klf6 Sox8-1 Nr4a1 Tcf7l2-1 Tcf7l2-2
MGTFmix
Stagia3
TATA
eGFP
IRES
AP
H
MG1(552bp)
TCF7L2 BS JASPAR score>15
MAF BS JASPAR score>16
241bp
290bp
MG1s (93bp)
Stagia3
TATA
eGFP
IRES
AP
P1 electroporation P21 harvest
MG1s
Stagia3
TATA
Cre-ER
Ai9
Electroporation of plasmids into P1 Ai9 retinas in vivo.
TM injection at P21. Harvest at P30.
pMG1s-GFP-IRES-AP pCAG-mCherry
H1 DAPI
H2
H3 GFP
H4 mCherry
pMG1s-CreER + TM
H5 DAPI
H6
H7 tdT
H8 Sox9

Fig. S2. The activities of MG CRMs in the mouse retina *in vivo* (associated with Fig 1).

(A) The experimental design. The CRM-containing reporter plasmids were co-electroporated into P1 WT mouse retinas *in vivo* with pCAG-mCherry plasmids, which served as electroporation efficiency controls. The retinas were harvested at P21. **(B-F)** The reporter plasmids for MG1 (B), MG3 (C), MG6 (D), MG10 (E) or MG12 (F) CRMs were individually co-electroporated into P1 WT mouse retinas *in vivo* with pCAG-BFP control plasmids. The retinas were harvested at P21. Red: BFP signals. Magenta: Sox9 antibody staining (labels MG cell bodies). Green: GFP signals. No immunostaining was performed to amplify the GFP signals. Scale bar: 20um. **(G)** The synthetic MGTFmix CRM was generated by stitching the TFBSs of MG-enriched TFs and cloned into the Stagia3 reporter plasmids. The activity of MGTFmix CRM was tested ex vivo in mouse retinas. Scale bar: 50um. **(H)** The shortened MG1 CRM (MG1s) is 93bp and contains the predicted TFBSs of Tcf7l2 and MAF (with highest JASPAR scores). (H1-H4) The pMG1s-GFP-IRES-AP reporter plasmids were co-electroporated into P1 WT mouse retinas *in vivo* with pCAG-mCherry plasmids. The retinas were harvested at P21. (H5-H8) The pMG1s-CreER plasmids were electroporated into P1 Ai9 homozygous mouse retinas *in vivo*. TM (tamoxifen) was administrated into these mice via IP injection at P21. The retinas were harvested at P30. Sox9: MG specific marker. Scale Bar: 20um.

Fig S3

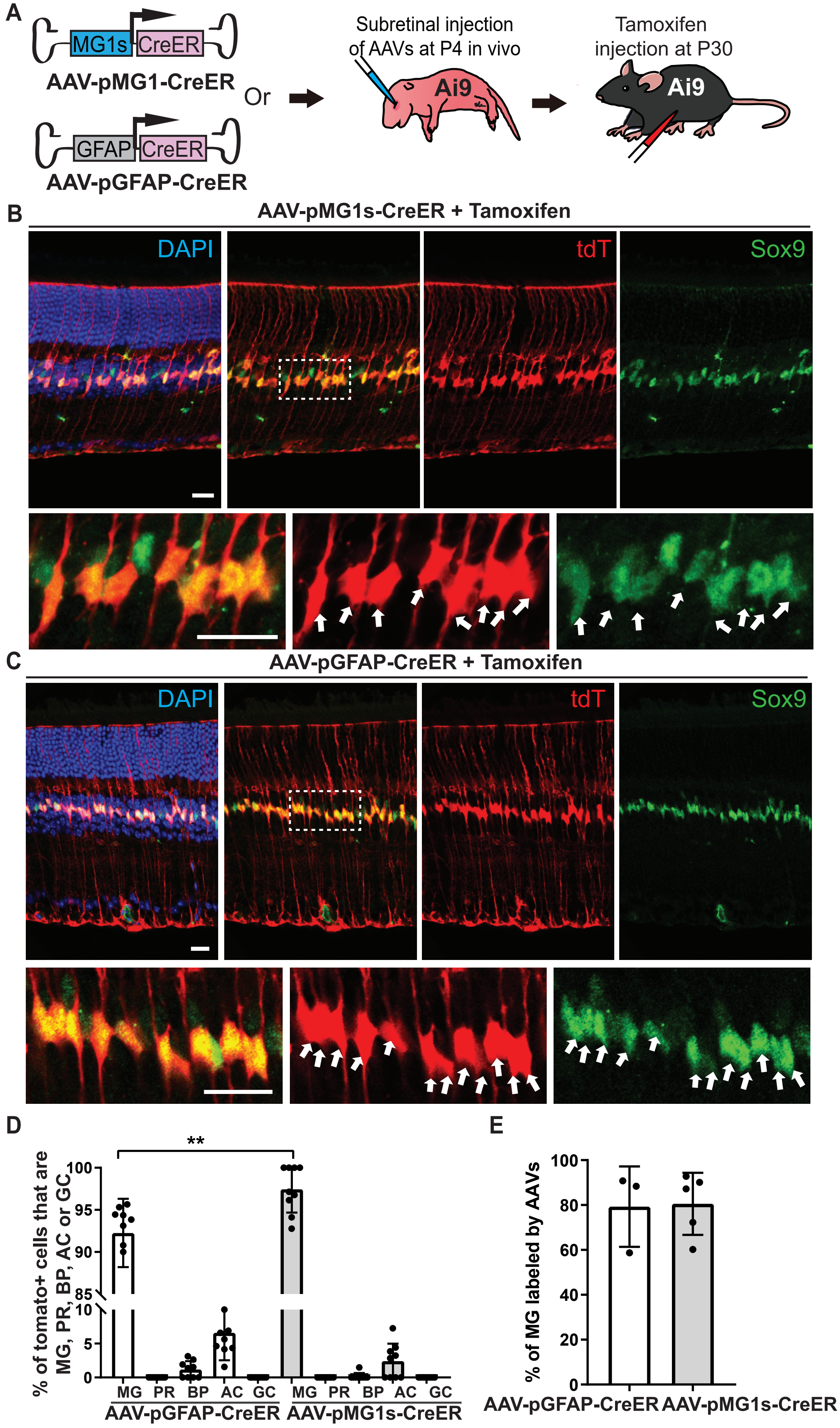
A
MG1s
CreER
AAV-pMG1-CreER
Or
GFAP
CreER
AAV-pGFAP-CreER
Subretinal injection
of AAVs at P4 in vivo
Ai9
Tamoxifen
injection at P30
Ai9
B
AAV-pMG1s-CreER + Tamoxifen
DAPI
tdT
Sox9
C
AAV-pGFAP-CreER + Tamoxifen
DAPI
tdT
Sox9
D
**
% of tomato+ cells that are MG, PR, BP, AC or GC
100
95
90
85
10
5
0
MG PR BP AC GC MG PR BP AC GC
AAV-pGFAP-CreER AAV-pMG1s-CreER
E
% of MG labeled by AAVs
100
80
60
40
20
0
AAV-pGFAP-CreER AAV-pMG1s-CreER

Fig. S3. AAV-pMG1s-CreER and AAV-pGFAP-CreER can both specifically and efficiently target retinal MG (associated with Fig 2).

(A) The experimental design. **(B & C)** The Ai9 homozygous mouse retinas transduced by AAV-pMG1s-CreER (B) or AAV-pGFAP-CreER (C) with AAV8 capsid. Green: Sox9 antibody staining labeled the nuclei of MG. tdT: tdTomato expression from the Ai9 reporter gene. High magnification view of the highlighted region was shown in panel B and C. White arrow: tdT+ MG. Scale bar: 20um. **(D-E)** The specificity (D) and efficiency (E) of AAV-pGFAP-CreER and AAV-pMG1s-CreER. Two-tailed student T-test. **: P<0.01. Mean±SD. N>=4 mice.

Fig S4

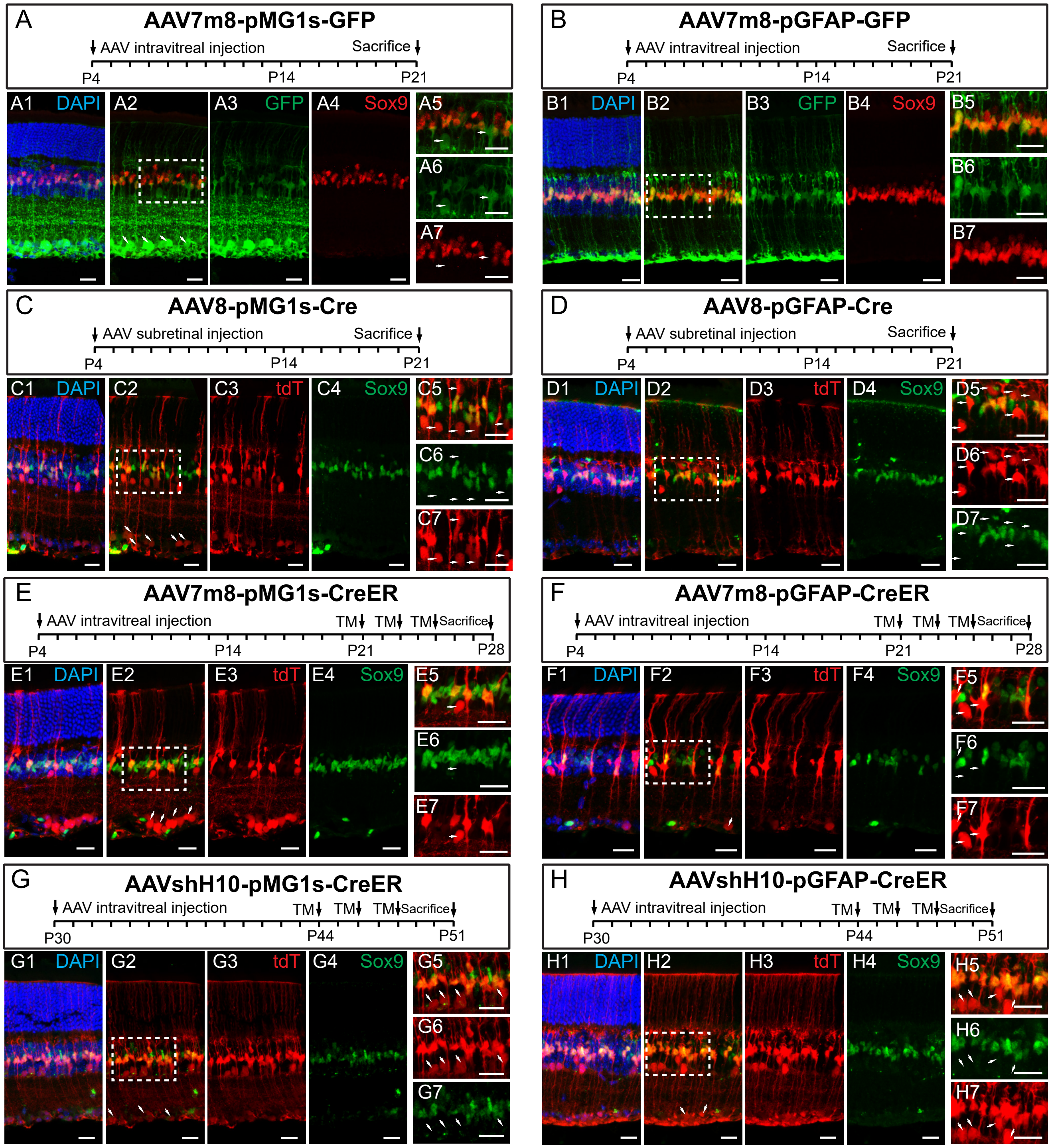

I

	Capsids	Injection route	Injection age	Specificity	Efficiency
AAV-pMG1s-GFP	AAV8	Subretinal	P4	~99%	~60%
	AAV7m8	Intravitreal	P4	<30%	N/A
AAV-pGFAP-GFP	AAV8	Subretinal	P4	~95%	~60%
	AAV7m8	Intravitreal	P4	~95%	~55%
AAV-pMG1s-Cre	AAV8	Subretinal	P4	~40%	~40%
AAV-pGFAP-Cre	AAV8	Subretinal	P4	~80%	~40%
AAV-pMG1s-Cre^{ER}	AAV8	Subretinal	P4	~97%	~80%
	AAV7m8	Intravitreal	P4	~39%	~30%
	AAVshH10	Intravitreal	P4 or P30	~60%	~60%
AAV-pGFAP-Cre^{ER}	AAV8	Subretinal	P4	~93%	~80%
	AAV7m8	Intravitreal	P4	~50%	~30%
	AAVshH10	Intravitreal	P4 or P30	~73%	~80%

Fig. S4. The specificity and efficiency of MG1s CRM and GFAP minipromoter in driving GFP/Cre/CreER expression with different capsids and routes of injection (associated with Fig 2).

(A-B) The AAV-pMG1s-GFP (A) or AAV-pGFAP-GFP (B) viruses with AAV7m8 capsids were injected into WT mouse retinas intravitreally. A5-7 or B5-7: high magnification view of the highlighted region in A2 or B2. Sox9: MG marker. White arrows: GFP+Sox9- cells (Non-specific labeling). **(C-D)** The AAV-pMG1s-Cre (C) or AAV-pGFAP-Cre (D) viruses with AAV8 capsids were injected into Ai9 homozygous mouse retinas subretinally. C5-7 or D5-7: high magnification view of the highlighted region in C2 or D2. White arrows: tdT (tdTomato)+Sox9- cells (Non-specific labeling). **(E-H)** The AAV-pMG1s-CreER (E&G) or AAV-pGFAP-CreER (F&H) viruses with AAV7m8 (E-F) or AAVshH10 (G-H) capsids were injected into Ai9 homozygous mouse retinas intravitreally. E5-7, F5-7, G5-7 or H5-7: high magnification view of the highlighted region in E2, F2, G2 or H2. White arrows: tdT (tdTomato)+Sox9- cells (Non-specific labeling). Scale bar (A-H): 20um. **(I)** Table summarizing the specificity and efficiency of AAVs with MG1 CRM or GFAP minipromoter. N>=4 animals.

Fig S5

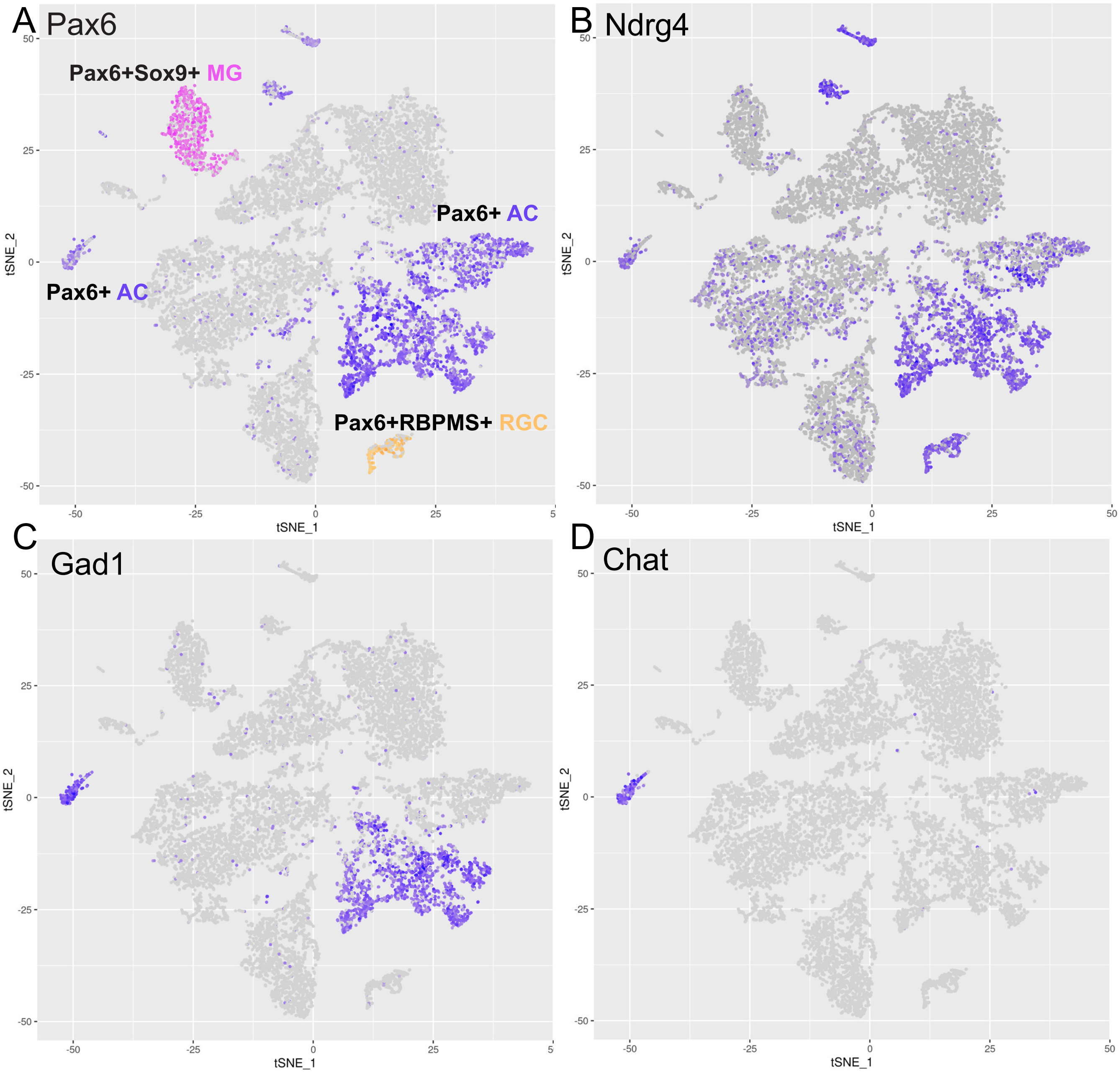
A Pax6
Pax6+Sox9+ MG
Pax6+ AC
Pax6+ AC
Pax6+RBPMS+ RGC
B Ndrg4
C Gad1
D Chat
tSNE_1
tSNE_2

Fig. S5. The expression pattern of *Ndrg4*, *Gad1* and *Chat* in the retina revealed by single cell RNA-seq data (Macosko et al. 2015) (associated with Fig 4).

(A) Pax6 is a pan-amacrine marker. $Pax6^{+}Sox9^{-}RBPMS^{-}$ cells were amacrine cells, which were highlighted in the TSNE plot. Cherry: Pax6+Sox9+ MG; Orange: Pax6+RBPMS+ RGCs; Purple: $Pax6^{+}Sox9^{-}RBPMS^{-}$ ACs. **(B-D)** The TSNE plots of Ndrg4, Gad1 or Chat positive cells.

Fig S6

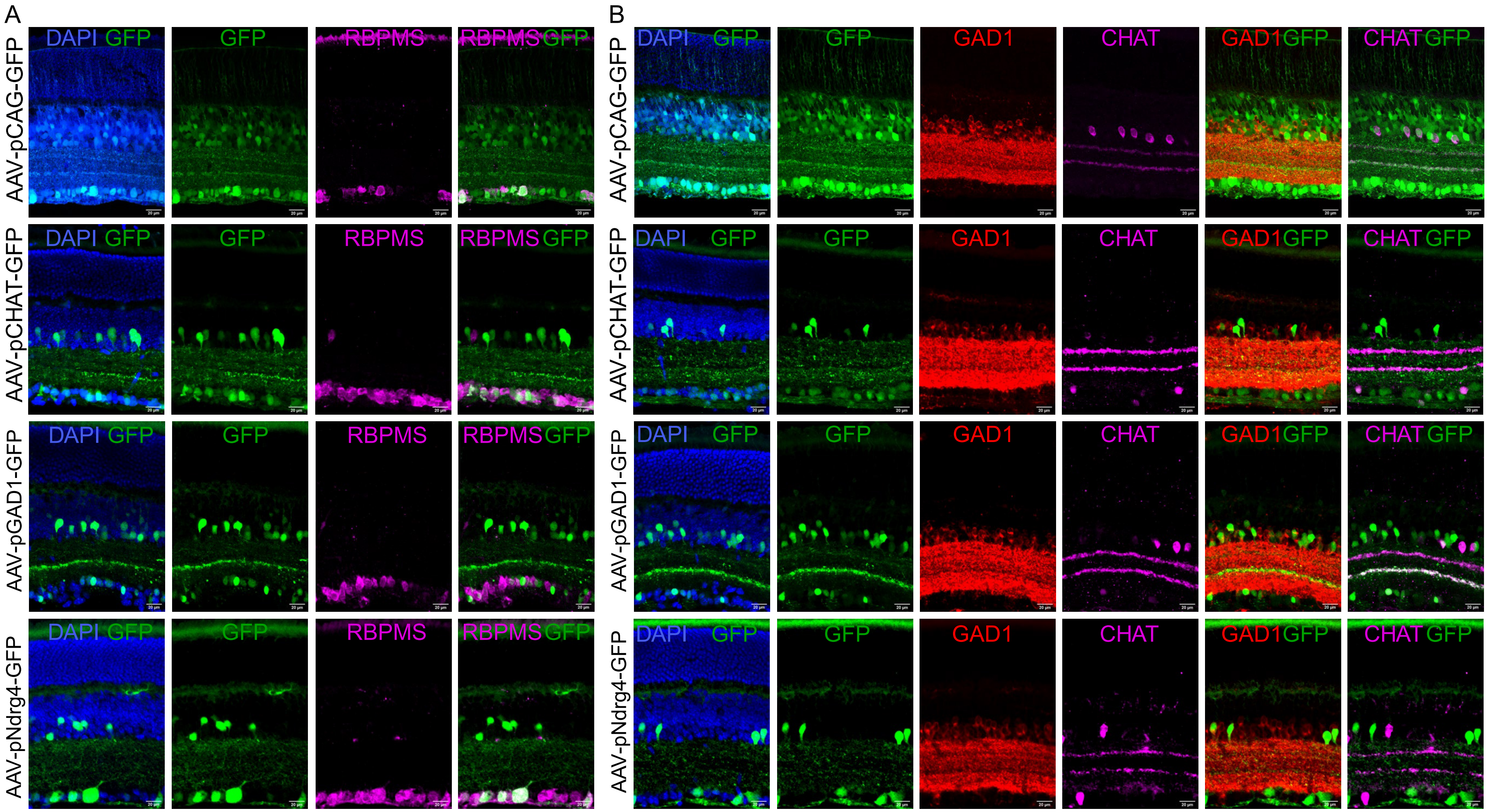
A
B
AAV-pCAG-GFP
AAV-pCHAT-GFP
AAV-pGAD1-GFP
AAV-pNdrg4-GFP
DAPI GFP
GFP
RBPMS
RBPMS GFP
GAD1
CHAT
GAD1GFP
CHAT GFP

Fig. S6. The cell types labeled by amacrine-targeting AAVs in the retina.

(A) Amacrine-targeting AAVs (AAV7m8 capsid) were injected intravitreally into WT mouse eyes at P4. The retinas were harvested at P21. The retina cryo-sections were co-stained with Pax6 and RBPMS antibodies. The Pax6 results were shown in Fig 4D. Here shows the RPBMS results. Magenta: RBPMS antibody staining labeled RGCs. Green: endogenous GFP signals from AAVs. **(B)** Amacrine-targeting AAVs mostly labeled Gad1+ CHAT- amacrine cells in INL. Green: endogenous GFP signals from AAVs. Red/Magenta: GAD1/CHAT antibody staining. Scale bar: 20um.

Fig S7

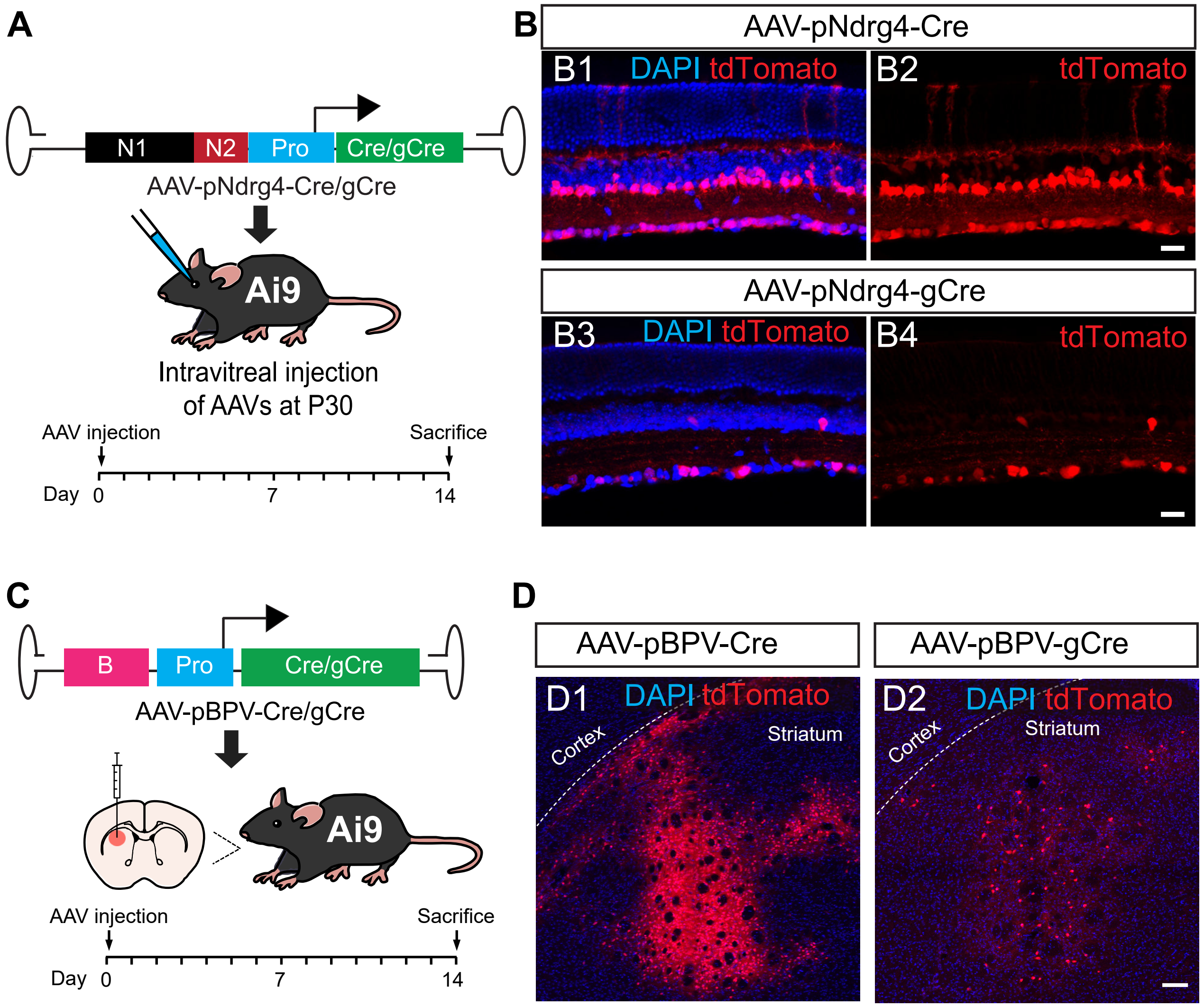
A
N1
N2
Pro
Cre/gCre
AAV-pNdrg4-Cre/gCre
Ai9
Intravitreal injection of AAVs at P30
AAV injection
Sacrifice
Day 0 7 14
B
AAV-pNdrg4-Cre
B1 DAPI tdTomato
B2 tdTomato
AAV-pNdrg4-gCre
B3 DAPI tdTomato
B4 tdTomato
C
B
Pro
Cre/gCre
AAV-pBPV-Cre/gCre
Ai9
AAV injection
Sacrifice
Day 0 7 14
D
AAV-pBPV-Cre
AAV-pBPV-gCre
D1 DAPI tdTomato
Cortex
Striatum
D2 DAPI tdTomato
Cortex
Striatum

Fig. S7. Desensitized gCre reduced non-specific recombination in the retina and brain.

(A) The AAV-pNdrg4-Cre or AAV-pNdrg4-gCre (with AAV7m8 capsid) viruses were intravitreally injected into the eyes of adult Ai9 homozygous mice at P30. Retinas were harvested 2 weeks later. **(B)** The activity of Cre/gCre in the retina, indicated by tdTomato expression. Scale bar: 20um. **(C)** The AAV-pBPV-Cre or AAV-pBPV-gCre (with AAV8 capsid) viruses were injected into adult Ai9 homozygous mouse brain. Brain tissue was harvested 2 weeks later. **(D)** The activity of Cre/gCre in the striatum, indicated by tdTomato expression. Scale bar: 100um.

Fig S8

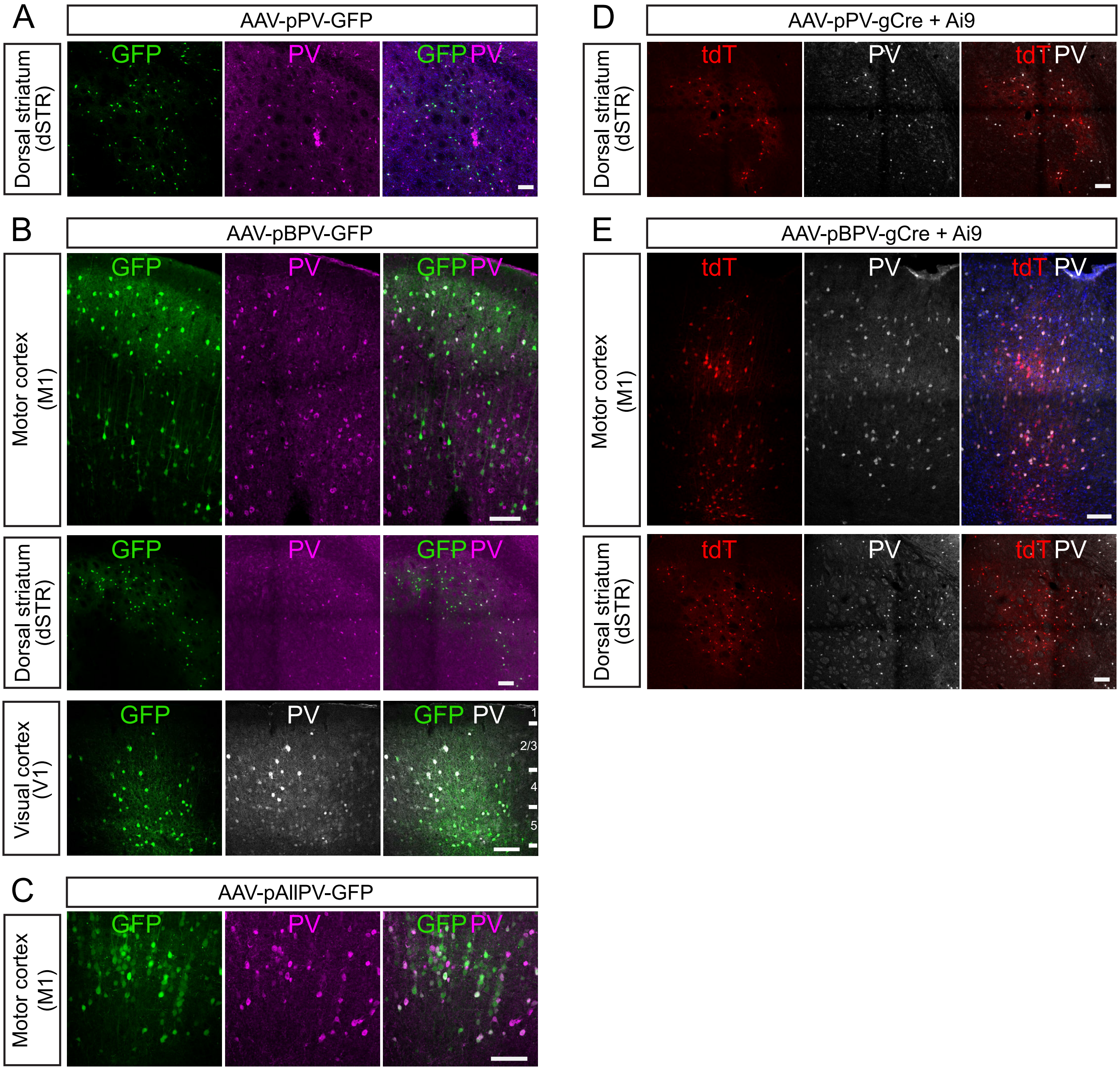
A
AAV-pPV-GFP
Dorsal striatum (dSTR)
GFP
PV
GFP PV
B
AAV-pBPV-GFP
Motor cortex (M1)
Dorsal striatum (dSTR)
Visual cortex (V1)
GFP
PV
GFP PV
1
2/3
4
5
C
AAV-pAllPV-GFP
Motor cortex (M1)
GFP
PV
GFP PV
D
AAV-pPV-gCre + Ai9
Dorsal striatum (dSTR)
tdT
PV
tdT PV
E
AAV-pBPV-gCre + Ai9
Motor cortex (M1)
Dorsal striatum (dSTR)
tdT
PV
tdT PV

Fig. S8. AAVs with synthetic CRMs targeted PV+ neurons in the brain.

(A) AAV-pPV-GFP transduced striatum. (B) AAV-pBPV-GFP transduced motor cortex (M1), visual cortex (V1) and dorsal striatum (dSTR). (C) AAV-pAllPV-GFP transduced motor cortex. (D) AAV-pPV-gCre transduced dorsal striatum. (E) AAV-pBPV-gCre transduced motor cortex (M1) and dorsal striatum (dSTR). AAV8 capsid was used for all AAVs. tdT: tdTomato. PV: antibody staining against PV. Scale bar: 100um.

Supplementary Table 1

The AAVs targeting PV+ cells in the brain

CRM name	Brain region	Genes driven by the CRM in AAVs	Specificity (%± sem)	Efficiency (% ± sem)	Reference
ProC17	V1	Fluorescent protein gene (e.g. GFP)	~90	N/A	Jüttner, 2019**
Scn1a-E2	S1	Fluorescent protein gene (e.g. GFP)	90.8 ± 1.1	75.7± 2.9	Vormstein-Schneider, 2020
	V1 cortex	Fluorescent protein gene (e.g. GFP)	88.1 ± 3.7	N/A	
	Cingulate cortex	Fluorescent protein gene (e.g. GFP)	80.2 ± 2.1	N/A	
	Subiculum	Fluorescent protein gene (e.g. GFP)	91.1 ± 2.1	N/A	
	CA1	Fluorescent protein gene (e.g. GFP)	80.5 ± 1.9	N/A	
	Striatum	Fluorescent protein gene (e.g. GFP)	54.8 ± 5.8	N/A	
	Baso-laterlal amygdala	Fluorescent protein gene (e.g. GFP)	38.1 ± 4.1	N/A	
	Substantia nigra reticulata	Fluorescent protein gene (e.g. GFP)	84.2 ± 1.1	N/A	
eHGT_023h	VISp	Fluorescent protein gene (e.g. GFP)	47 ± 4	N/A	Mich, 2021
eHGT_064h	VISp	Fluorescent protein gene (e.g. GFP)	50 ± 6	N/A	
eHGT_079h	VISp	Fluorescent protein gene (e.g. GFP)	87 ± 12	N/A	
eHGT_082h	VISp	Fluorescent protein gene (e.g. GFP)	93 ± 3	N/A	
eHGT_128h	VISp	Fluorescent protein gene (e.g. GFP)	93 ± 5	N/A	
eHGT_140h	VISp	Fluorescent protein gene (e.g. GFP)	99 ± 1	N/A	
AIIPV	M1	Fluorescent protein gene (e.g. GFP)	35		This manuscript
	Striatum	Fluorescent protein gene (e.g. GFP)	67.45 ± 4.9	90 ± 3.1	
BPV	M1	Fluorescent protein gene (e.g. GFP)	43	N/A	
	V1	Fluorescent protein gene (e.g. GFP)	88	N/A	
	Striatum	Fluorescent protein gene (e.g. GFP)	42	N/A	
	M1	gCre	59	N/A	
	Striatum	gCre	33	N/A	
pPV	M1	Fluorescent protein gene (e.g. GFP)	78.5 ± 4.9	77.8 ± 3.7	
	Striatum	Fluorescent protein gene (e.g. GFP)	44	N/A	
	M1	gCre	71.2 ± 12.3	62.1 ± 2.1	
	Striatum	gCre	24	N/A	

**: This result was showed in the preprint at BioRxiv but was removed from the published version at Nature Neurosicence.
